# Utilization and prescription patterns of traditional Chinese medicine for patients with hepatitis C in Taiwan: a population-based study

**DOI:** 10.1186/s12906-016-1379-3

**Published:** 2016-10-21

**Authors:** Chia-Yu Liu, Jui-Ying Chu, Jen-Huai Chiang, Hung-Rong Yen, Chung-Hua Hsu

**Affiliations:** 1 Institute of Traditional Medicine, School of Medicine, National Yang-Ming University, Taipei, Taiwan; 2 Department of Chinese Medicine, Branch of Linsen and Chinese Medicine, Taipei City Hospital, Taipei, Taiwan; 3 School of Traditional Chinese Medicine, College of Medicine, Chang Gung University, Taoyuan, Taiwan; 4 Research Centre for Chinese Medicine & Acupuncture, China Medical University, Taichung, Taiwan; 5 Health Data Management Office, China Medical University Hospital, Taichung, Taiwan; 6 Graduate Institute of Integrated Medicine, College of Chinese Medicine, China Medical University, Taichung, Taiwan; 7 Department of Chinese Medicine, China Medical University Hospital, 2 Yude Rd, North District, Taichung, 404 Taiwan; 8 School of Chinese Medicine, College of Chinese Medicine, China Medical University, Taichung, Taiwan

**Keywords:** Complementary and alternative medicine, Hepatitis C, National Health Insurance Research Database, Traditional Chinese medicine

## Abstract

**Background:**

To characterize the utilization of Traditional Chinese Medicine (TCM) among patients with hepatitis C (HC).

**Methods:**

This study examined datasets from the National Health Insurance Research Database in Taiwan. One cohort, including one million patients randomly sampled from the beneficiaries of the National Health Insurance Programme from January 1 to December 31 in 2010, was chosen for this analysis. People who had at least three outpatient or inpatient records and had been diagnosed with hepatitis C virus infection from 2000 to 2010 were defined as patients with HC. Patients with HC who had at least one TCM outpatient clinical record from 2000 to 2010 were defined as TCM users (*N* = 5,691), whereas patients with no TCM outpatient records were defined as non-TCM users (*N* = 2,876). The demographic data, treatment modalities and disease distributions of TCM users were analysed.

**Results:**

Overall, 66.4 % of the patients with HC had used TCM from 2000 to 2010. Of the TCM users, 54.1 % were female. The utilization rate of TCM increased with age and peaked in the age group of those 40 − 64 years old. Herbal remedies (52.4 %) were the most commonly used agents, followed by combination therapy (46.4 %) and acupuncture alone (1.2 %). Patients who had more extrahepatic diseases and were taking more antiviral agents tended to visit TCM clinics. Jia-Wei-Xiao-Yao-San and Dan-Shen (*Salvia miltiorrhiza*) were the most commonly used formula and single herb, with 88,124 person-days and 59,252 person-days, respectively.

**Conclusions:**

Our nationwide population-based study revealed a high prevalence and specific usage patterns of TCM in patients with HC in Taiwan.

## Background

The use of complementary and alternative medicine (CAM) is expanding throughout the world [1, 2]. According to the World Health Organization (W.H.O.), CAM includes Ayurveda, traditional Chinese medicine (TCM) and Unani medicine. TCM is widely used in East Asia [3]. Of the twenty-three million people in Taiwan, 29.1 % used TCM to treat disease in 2014, whereas up to three-fourths of South Korean adults utilized TCM to treat a specific disease [4].

Hepatitis C virus (HCV), which affects 180 million people globally, is a leading cause of chronic hepatitis, cirrhosis, and hepatocellular carcinoma [5]. Conventional anti-viral therapy consisting of pegylated interferon and ribavirin is associated with many intolerable side effects and low response rates in some patients’ genotypes [6]. Therefore, patients often seek for alternative treatments to promote healing and obtain support [7]. However, the communication regarding CAM between patients and physicians is relatively poor [8, 9], and the non-disclosure rate is higher than 70 %. Many physicians feel uncomfortable discussing CAM because of their limited knowledge of the subject. Patients avoid discussing CAM with their doctors because they fear receiving a negative response [10]. To provide holistic care to patients with hepatitis C (HC), physicians should understand the approaches used by patients for symptom relief and health maintenance. In the United States, 80 % of patients with HC used CAM according to a report from 2007 [11]. Compared with patients with fatty liver disease, patients with HC were approximately 3 times more likely to use CAM [12]. Because there has been growing interest in using CAM to treat populations with HC worldwide [13], information about CAM and comprehensive studies on its prevalence, usage patterns, efficacy and safety are important.

TCM, defined by the National Centre for Complementary and Integrative Health (NCCIH, U.S.A.) as an entire medical system of CAM, is a well-established medical system that has been used for more than 2,000 years. TCM is commonly used by the Chinese population, as well as by those in many other countries [14], including Taiwan [15]. In Taiwan, TCM has been reimbursed by the National Health Insurance (NHI) programme since 1996. As of 2014, 99.9 % of Taiwan’s population were enrolled in the NHI. All of the claims data have been collected in the National Health Insurance Research Database (NHIRD). According to the NHI programme guidelines, TCMs are only provided for outpatient care including Chinese herbal prescriptions, acupuncture, and traumatology manipulative therapy. The utilization prevalence of TCM in Taiwan ranges from 19.8 % to 77.9 % for many diseases including colon cancer [16], liver cancer [17], osteoporosis [18], and type II diabetes mellitus [19]. However, the utilization and prescription patterns of TCM in HC are lacking.

To characterize the utilization patterns and trends in TCM usage among patients with HC, we analysed a cohort of one million randomly sampled beneficiaries from the NHIRD in 2010. The results of this study should provide valuable information for physicians and for patients with HC.

## Methods

### Data source

As previously described in detail [20], all TCM services covered under the NHI are provided only in ambulatory clinics. In Taiwan, TCM physicians (those who have received a series of training in Chinese or both Chinese and Western medicine, all of whom must pass national licensing examinations and complete residency training programmes in hospitals) are requested to make diagnoses based on the International Classification of Disease, 9th Revision, Clinical Modification (ICD-9-CM) coding [16]. This study chose one cohort for the analysis, which included one million patients randomly sampled from the beneficiaries of the NHI programme in 2010. The NHIRD contains information on the medical care facilities, physician specialties, and patients’ gender, dates of birth, dates of visit, masked identification numbers, prescriptions, management and diagnosis codes in the ICD-9-CM. A maximum of three diagnostic codes were listed in the NHIRD, and all the diagnoses were analysed in our study. This study was approved by the Research Ethics Committee of the Taipei City Hospital (TCHIRB-10406112-E).

### Study subjects

**Fig. 1:**
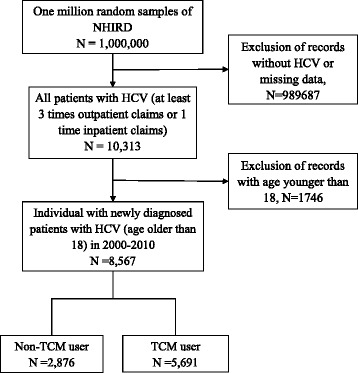
Flow chart of subjects in the one million randomly selected sample from the National Health Insurance Research Database (NHIRD) from 2000 to 2010 in Taiwan

The study subjects were selected from a random sample of one million individuals in 2010 in the following manner (Fig. 1): People who had at least three outpatient or inpatient records and had been diagnosed with HCV from January 1, 2000, to December 31, 2010, were defined as patients with HC (ICD-9-CM codes 07054, 0707, 07041, 07044, 07051, V0262). Based on this criteria, there were 8,567 patients older than 18 years old with a new HCV infection diagnosed starting from the index date of January 1, 2000. Patients with HC who had at least one TCM clinical record from 2000 to 2010 were defined as TCM users (*N* = 5,691), whereas those who had no TCM records during the same period were defined as non-TCM users (*N* = 2,876). All study subjects were followed until December 31, 2011.

### Statistical analysis

The data were analysed using SAS software program, version 9.4 (SAS Institute Inc., Cary, NC, U.S.A.). A univariate analysis was used to compare the TCM users with the non-TCM users. Chi-squared test was performed to assess the relationships between the categorical variables and to examine the differences between TCM users and non-users. Person-years for the follow-up period were calculated for each patient until diagnosis of multisite diseases, censor or December 31, 2011. The person-years and overlapping confidence interval (CI) were calculated to assess incidence density rates. To compare the study cohort to the comparison cohort rate, ratios were examined using a Poisson regression model. Moreover, we estimated the adjusted hazard ratios using Cox proportional hazards model. A *P* value <0.05 was considered statistically significant.

## Results

### Demographic characteristics of the TCM users with HC

Of the patients with HC, 66.4 % (*N* = 5,691) had previously used TCM (Table 1). In the TCM users, 54.1 % were female, and this differed from the percentage in non-TCM users, which was only 39.9 %. In both TCM users and non-users, the highest proportion of patients was in the age group from 40 to 64 years old. Regarding the comorbidities of patients with HC, the incidence of most diseases, including osteoarthritis, sicca syndrome, thyroid disorders, chronic obstructive pulmonary disease, and hepatitis B, were higher in TCM users, except for diabetes mellitus, which had a similar incidence in both groups. In contrast, the incidence of hepatocellular carcinoma, liver cirrhosis, and alcohol-related diseases were higher in non-TCM users.

**Table 1:** Demographic characteristics of the patients with hepatitis C in Taiwan in 2000-2011

Variable	Non-TCM		TCM		*p* value
	*N* = 2876 (33.57 %)		*N* = 5691 (66.43 %)		
	*n*	%	*n*	%	
Sex					<0.0001
Female	1148	39.92	3081	54.14	
Male	1728	60.08	2610	45.86	
Age at baseline					<0.0001
18−39	364	12.66	945	16.61	
40−64	1451	50.45	3395	59.66	
≥ 65	1061	36.89	1351	23.74	
Mean (STD)	58.34	14.85	53.92	13.97	
Urbanization^a^					<0.0001
1 (highest)	574	19.96	1215	21.35	
2	731	25.42	1652	29.03	
3	447	15.54	939	16.50	
4+ (lowest)	1124	39.08	1885	33.12	
Co-morbidity					
Hepatitis B	570	19.82	1356	23.83	<0.0001
Liver cirrhosis	989	34.39	1618	28.43	<0.0001
hepatocellular carcinoma	535	18.60	898	15.78	0.0009
Alcohol-related disease	294	10.22	477	8.38	0.0049
Chronic obstructive pulmonary disease	508	17.66	1402	24.64	<0.0001
Diabetes Mellitus	1046	36.37	2143	37.66	0.2449
Thyroid disorders	145	5.04	631	11.09	<0.0001
Rheumatoid arthritis	89	3.09	400	7.03	<0.0001
Osteoarthritis	953	33.14	2922	51.34	<0.0001
Depression	174	6.05	650	11.42	<0.0001
Sicca syndrome	78	2.71	423	7.43	<0.0001
Charlson comorbidity index score					<0.0001
0	1203	41.83	3024	53.14	
1	377	13.11	769	13.51	
More than 2	1296	45.06	1898	33.35	
Anti-viral or hepatoprotective agents					
Interferon alfa	392	13.63	974	17.11	<0.0001
Ribavirin	391	13.60	973	17.10	<0.0001
Silymarin	1916	66.62	4204	73.87	<0.0001
Times of visits (mean, SD)	15.61	17.18	21.23	23.18	<0.0001

^a^The townships within which subjects registered for insurance were grouped into 4 levels of urbanization, based on a score calculated by incorporating variables indicating population density (people/km2), and population ratio of different educational levels, population ratio of elderly, population ratio of people of agriculture workers and the number of physicians per 100,000 people

To examine the frequency of utilization of Western medicine, we analysed the claims data according to the antiviral or hepatoprotective agents used (Table 1). A higher percentage of TCM users than non-TCM users had ever used these agents to control hepatitis C (all *p*-values < 0.001). TCM users tended to use interferon-alpha, ribavirin, and silymarin to treat HCV (17.1 %, 17.1 %, and 73.9 %, respectively).

With regard to TCM outpatient visits (Table 2), herbal remedies (52.4 %) were the most commonly used therapeutic approach, followed by combined therapy (46.4 %) and acupuncture alone (1.12 %). As for the frequency of visits, the majority of patients visited TCM clinics fewer than three times (74.01 %). Only 16.64 % of the patients visited TCM clinics more than six times.

**Table 2:** Distribution of Chinese medicine according to type of Chinese Medicine treatment received in patients with hepatitis C, stratified by number of outpatients visits

Number of TCM visits (times/per year)	Only Chinese herbal remedies	Only Acupuncture or traumatology	Combination both treatment	Total of TCM (*N* = 5691)
	*N* = 2984 (52.43 %)	*N* = 64 (1.12 %)	*N* = 2643 (46.44 %)	
1-3	2423 (81.20 %)	64 (100 %)	1725 (65.27 %)	4212 (74.01 %)
4-6	212 (7.10 %)	0	320 (12.11 %)	532 (9.35 %)
>6	349 (11.70 %)	0	598 (22.63 %)	947 (16.64 %)

### The prevalence of hepatitis B, liver cirrhosis, alcohol-related disease, chronic obstructive pulmonary disease and diabetes mellitus was lower in TCM users than non-TCM users in Taiwanese HC patients

After adjusting for the frequency of outpatient and inpatient visits, Charlson comorbidity index score, and type of service used - only herbal medicine, only acupuncture or a combination - we calculated the disease hazard ratio (HR) of TCM users versus non-TCM users to compare the prevalence of comorbidities between them. We found that TCM users tended to have certain comorbidities less frequently than non-TCM users (Table 3). TCM users had a 0.55 times lower HR of having hepatitis B than non-TCM users (95 % confidence interval 0.46 − 0.66). Patients who received TCM treatment also had a lower ratio for liver cirrhosis (HR 0.42, with 95 % CI 0.37 − 0.48) and alcohol-related disease (HR: 0.31 for the 40−64 age group and 0.41 overall). The prevalence of chronic obstructive pulmonary disease was also lower in the TCM users (HR: 0.59 overall). Diabetes mellitus also showed a lower incidence in TCM users than non-TCM users overall and in each age group (HR: 0.48, 0.40, 0.46 and 0.64 for the sample overall and for the <40, 40–64, and >60 age groups, respectively). However, after adjusting for times of visit, Charlson comorbidity index score, and type of TCM service used, the hazard ratio of comorbidities such as sicca syndrome, rheumatoid arthritis, depression, and thyroid disorders was less than 1, with no significance.

**Table 3:** Incidence rate ratio for common disease between non-TCM and TCM user in different age groups

Age groups (year-old)	Non-TCM user			TCM user			IRR (95 % CI)	Adjusted HR^b^
Disease (ICD-9-CM)	*N* (%)	Person-years	IR^a^	*N* (%)	Person-years	IR^a^		(95 % CI)
Hepatitis B (70.2, 070.3, V02.61)								
All	570(19.82)	13085	43.56	1356(23.83)	39350	34.46	0.79(0.72−0.87)***	0.55(0.46−0.66)***
18−39	88(24.18)	1901	46.3	239(25.29)	7189	33.24	0.72(0.56−0.92)**	0.41(0.25−0.69)***
40−64	327(22.54)	6923	47.23	842(24.80)	23900	35.23	0.75(0.66−0.85)***	0.48(0.37−0.6)***
≥ 65	155(14.61)	4261	36.37	275(20.36)	8260	33.29	0.92(0.75−1.11)	0.82(0.58−1.17)
Liver cirrhosis (571.2, 571.5, 571.6, 572.2, 572.3, 572.4, 572.8, 573.0)								
All	989(34.39)	13085	75.58	1618(28.43)	39350	41.12	0.54(0.50−0.59)***	0.42(0.37−0.48)***
18−39	45(12.36)	1901	23.68	108(11.43)	7189	15.02	0.63(0.45−0.90)*	0.43(0.25−0.73)**
40−64	489(33.70)	6923	70.63	951(28.01)	23900	39.79	0.56(0.51−0.63)***	0.39(0.33−0.46)***
≥ 65	455(42.88)	4261	106.77	559(41.38)	8260	67.67	0.63(0.56−0.72)***	0.54(0.44−0.66)***
hepatocellular carcinoma (155)								
All	535(18.60)	13085	40.89	898(15.78)	39350	22.82	0.56(0.50−0.62)***	0.78(0.45−1.36)
18−39	6(1.65)	1901	3.16	33(3.49)	7189	4.59	1.45(0.61−3.47)	1.25(0.12−12.75)
40−64	242(16.68)	6923	34.96	514(15.14)	23900	21.51	0.62(0.53−0.72)***	0.98(0.43−2.21)
≥ 65	287(27.05)	4261	67.35	351(25.98)	8260	42.49	0.63(0.54−0.74)***	0.72(0.29−1.75)
Alcohol-related disease (291, 303.0, 303.9, 305.0, 571.0, 571.1, 571.3)								
All	294(10.22)	13085	22.47	477(8.38)	39350	12.12	0.54(0.47−0.62)***	0.41(0.32−0.51)***
18−39	65(17.86)	1901	34.2	124(13.12)	7189	17.25	0.50(0.37−0.68)***	0.37(0.24−0.58)***
40−64	185(12.75)	6923	26.72	290(8.54)	23900	12.13	0.45(0.38−0.55)***	0.31(0.23−0.42)***
≥ 65	44(4.15)	4261	10.33	63(4.66)	8260	7.63	0.74(0.50−1.09)	0.61(0.33−1.16)
Chronic obstructive pulmonary disease (491, 492)								
All	508(17.66)	13085	38.82	1402(24.64)	39350	35.63	0.92(0.83−1.12)	0.59(0.50−0.70)***
18−39	27(7.42)	1901	14.21	120(12.70)	7189	16.69	1.18(0.77−1.78)	0.94(0.45−1.97)
40−64	198(13.65)	6923	28.6	786(23.15)	23900	32.89	1.15(0.98−1.34)	0.67(0.52−0.86)**
≥ 65	283(26.67)	4261	66.41	496(36.71)	8260	60.05	0.90(0.78−1.05)	0.69(0.53−0.89)**
Diabetes Mellitus (250)								
All	1046(36.37)	13085	79.94	2143(37.66)	39350	54.46	0.68(0.63−0.73)***	0.48(0.42−0.55)***
18−39	55(15.11)	1901	28.94	151(15.98)	7189	21	0.73(0.53−0.99)*	0.40(0.22−0.71)**
40−64	572(39.42)	6923	82.62	1358(40.00)	23900	56.82	0.69(0.62−0.76)***	0.46(0.38−0.54)***
≥ 65	419(39.49)	4261	98.32	634(46.93)	8260	76.75	0.78(0.69−0.88)***	0.64(0.51−0.8)***
Thyroid disease (240, 241, 242, 244)								
All	145(5.04)	13085	11.08	631(11.09)	39350	16.04	1.44(1.21−1.73)***	0.90(0.67−1.21)
18−39	21(5.77)	1901	11.05	105(11.11)	7189	14.6	1.32(0.83−2.11)	1.33(0.54−3.28)
40−64	79(5.44)	6923	11.41	418(12.31)	23900	17.49	1.53(1.21−1.95)***	0.9(0.6−1.36)
≥ 65	45(4.24)	4261	10.56	108(7.99)	8260	13.07	1.24(0.87−1.75)	0.92(0.53−1.58)
Rheumatoid arthritis (714)								
All	89(3.09)	13085	6.8	400(7.03)	39350	10.17	1.49(1.19−1.88)***	1.18(0.82−1.69)
18−39	11(3.02)	1901	5.79	42(4.44)	7189	5.84	1.01(0.52−1.96)	0.88(0.28−2.78)
40−64	45(3.10)	6923	6.5	266(7.84)	23900	11.13	1.71(1.25−2.35)***	1.38(0.81−2.36)
≥ 65	33(3.11)	4261	7.74	92(6.81)	8260	11.14	1.44(0.97−2.14)	1.24(0.69−2.22)
Osteoarthritis (715)								
All	953(33.14)	13085	72.83	2922(51.34)	39350	74.26	1.02(0.95−1.10)	0.75(0.66−0.86)***
18−39	46(12.64)	1901	24.2	215(22.75)	7189	29.91	1.24(0.90−1.70)	1.2(0.64−2.27)
40−64	427(29.43)	6923	61.68	1777(52.34)	23900	74.35	1.21(1.08−1.34)***	0.85(0.71−1.03)
≥ 65	480(45.24)	4261	112.64	930(68.84)	8260	112.59	1.00(0.90−1.12)	0.82(0.67−1)
Depression (296.2, 296.3, 296.5, 296.6, 305.8, 311, v790, 290.13)								
All	174(6.05)	13085	13.3	650(11.42)	39350	16.52	1.24(1.05−1.47)*	0.78(0.61−1.01)
18−39	31(8.52)	1901	16.31	128(13.54)	7189	17.8	1.09(0.74−1.62)	0.76(0.42−1.38)
40−64	86(5.93)	6923	12.42	382(11.25)	23900	15.98	1.29(1.02−1.63)*	0.73(0.52−1.04)
≥ 65	57(5.37)	4261	13.38	140(10.36)	8260	16.95	1.27(0.93−1.72)	0.89(0.54−1.48)
Sicca syndrome (370.33, 710.2)								
All	78(2.71)	13085	5.96	423(7.43)	39350	10.75	1.80(1.42−2.30)***	1.27(0.86−1.89)
18−39	4(1.10)	1901	2.1	42(4.44)	7189	5.84	2.78(1.00−7.74)	-
40−64	40(2.76)	6923	5.78	266(7.84)	23900	11.13	1.93(1.38−2.69)***	1.14(0.67−1.94)
≥ 65	34(3.20)	4261	7.98	115(8.51)	8260	13.92	1.75(1.19−2.56)**	1.71(0.93−3.15)

^**a**^ *IR* incidence rate, per 1000 person-years, *IRR* incidence rate ratio

*:<0.05; **:<0.01; *** *p* < 0.001

^**b**^Hazard Ratio adjusted for times of outpatient and inpatient visit, Charlson comorbidity index score and type of service used - only herbal medicine, only acupuncture and combination of them

### Frequency distribution of disease categories in TCM versus non-TCM visits

To delineate the frequency distributions of the disease categories (as the reasons for visits) for the TCM and non-TCM visits, we analysed the ICD-9-CM codes from the claims data (Table 4). There was a significant difference in the disease distributions between the TCM and non-TCM users (*P* < 0.0001). Among all of the visits, infectious diseases (99.68 %), which included viral hepatitis, were the most common reasons that TCM users visited TCM clinics. Digestive system diseases (99.61 %), which included chronic liver disease, were the second most common reason that TCM users visited TCM clinics. Symptoms/signs and ill-defined conditions (97.96 %) and respiratory system diseases (97.93 %) accounted for the third and fourth disease categories, respectively, followed by diseases of the musculoskeletal system and connective tissue (93.89 %) and injuries (90.77 %). For non-TCM users, infectious diseases (99.51 %) were the most common reason for visiting Western medical clinics, followed by digestive system diseases (97.25 %) and symptoms/signs and ill-defined conditions (89.33 %). When TCM users required medical services, their utilization patterns were similar to those of non-TCM users.

**Table 4:** The distribution of TCM and non-TCM user by major disease categories /diagnosis in patients with hepatitis C

Disease (ICD-9-CM)	Non-TCM user (*N* = 2876)		TCM user (*N* = 5691)		*p* value
	*n*	%	*n*	%	
Infectious and parasitic disease (001−139)	2862	99.51	5673	99.68	0.2218
Neoplasms (140−239)	1289	44.82	3121	54.84	<0.0001
Malignant(140−208)	824	28.65	1491	26.20	0.0158
Benign (210−229)	597	20.76	2152	37.81	<0.0001
Endocrine, nutritional and metabolic disease and immunity disorder (240−279)	1863	64.78	4133	72.62	<0.0001
Blood and blood-forming organs (280−289)	843	29.31	1876	32.96	0.0006
Mental disorder (290−319)	1206	41.93	3360	59.04	<0.0001
Nervous system (320−389)	2087	72.57	5120	89.97	<0.0001
Circulatory system (390−459)	2123	73.82	4410	77.49	0.0002
Respiratory system (460−519)	2548	88.60	5573	97.93	<0.0001
Digestive system (520−579)	2797	97.25	5669	99.61	<0.0001
Genitourinary system (580−629)	1760	61.20	4485	78.81	<0.0001
Complications of pregnancy, childbirth and the puerperium (630−676)	19	0.66	178	3.13	<0.0001
Skin and subcutaneous tissue (680−709)	1958	68.08	4830	84.87	<0.0001
Musculoskeletal system and connective tissue (710−739)	2091	72.71	5343	93.89	<0.0001
Congenital anomalies (740−759)	126	4.38	511	8.98	<0.0001
Certain conditions originating in the perinatal period (760−779)	13	0.45	43	0.76	0.0997
Symptoms, signs and ill-defined conditions (780−799)	2569	89.33	5575	97.96	<0.0001
Injury and poisoning (800−999)	1997	69.44	5166	90.77	<0.0001

### The most commonly used TCM prescriptions

To comprehensively understand the TCM prescriptions, including the formulas and herbs, we analysed the claims data, and the results are shown in Table 5. Of the 10 most common formulas of TCM used by patients with HC, Jia-wei-xiao-yao-san (88,124 person-days) was the most commonly used. Xiao-chai-hu-tang (39,837 person-days) and Long-dan-xie-gan-tang (36,293 person-days) accounted for the second and third most commonly used formulas, respectively. With regard to the single herbs used for TCM by patients with HC, Dan-shen (59,252 person-days) was the most common. Yan-hu-suo (41,875 person-days) and Huang-qin (35,273 person-days) were the second and third most commonly used herbs, respectively.

**Table 5:** Most common Chinese herbs and formula prescribed for patients with hepatitis C

Prescription name (in Chinese)	Ingredients	Therapeutic action and Indication	Number of person-days	Average daily dose (g)	Average duration for prescription (days)
Single herb					
Dan-shen	*Salvia miltiorrhiza* Bunge	H & E: Activate blood and resolve stasis anti-fibrosis, antihepatocarcinoma, anti-diabetic, lipid-lowering	59252	2.5	10.1
Yan-hu-suo	*Corydalis yanhusuo*	H & E: Activate blood, promote flow of qi, and alleviate pain Also used in peptic ulcer	41875	3	7.1
Huang-qin	*Scutellaria baicalensis* Georgi	H: Clear heat and drain fire Anti-inflammation	35273	2.2	8.1
Yin-chen-hao	*Artemisia capillaris* Thunb	H: Excrete dampness and alleviate jaundice Anti-fibrosis	33357	2.4	10.1
Da-huang	*Rheum officinale* Baill	E: Clear heat and drain fire Anti-tumor	32110	1.1	7.9
Huang-qi	*Astragalus membranaceus*	H&E: Qi-tonifying/ restore energy Anti-cancer	30086	2.1	8.8
Bei-mu	*Fritillariae thunbergii* Bulbus	E: Clear heat and resolve phlegm Also used in peptic ulcer and asthma	29404	2.7	7.1
Ge-gen	*Pueraria thomsonii* Benth	E: Release exterior and cure heat Also used in ischemic heart disease	29143	2	7.4
Ye-jiao-teng	*Polygonum multiflorum* Thunb.	E: Nourish heart and induce tranquilization Also used in menopausal syndrome	28559	3.3	8.7
Hai-piao-xiao	*Sepiella maindronide* Rochebrune	E: Restrain acidity and alleviate pain	27940	2.5	8.4
Formulae		Also used in peptic ulcer			
Jia-wei-xiao-yao-san	*Glycyrrhiza uralensis* Fisch. *Angelica sinensis*, *Atractylodes macrocephala*, *Bupleurum chinense*, *Gardenia jasminoides*, *Mentha haplocalyx*, *Paeonia lactiflora*, *Paeonia suffruticosa*, *Poria cocos*, *Zingiber officinale*	H & E: Harmonize liver and release spleen; Also used in thyroid disorders	88124	7.5	9.3
Xiao-chai-hu-tang	*Bupleurum chinense*, *Scutellaria baicalensis* Georgi, *Pinellia ternata* (Thunb.) Makino, *Panax ginseng* C.A. Mey, *Glycyrrhiza uralensis* Fisch, *Zingiber officinale*, *Zizyphus jujuba*	H: Regulate exterior and interior Qi activity by balancing between yin and yang; Antihepatocarcinoma	39837	6.7	7.9
Long-dan-xie-gan-tang	*Gentiana scabra* Bge, *Scutellaria baicalensis* Georgi, *Gardenia jasminoides* Ellis, *Alisma orientalis*, *Akebia quinata* (Houtt.) Decne., *Plantago asiatica* L., *Angelica sinensis*, *Rehmannia glutinosa* (Gaert.) Libosch., *Bupleurum chinense*, *Glycyrrhiza uralensis* Fisch.	H: Purge fire in the liver and gallbladder, clear away damp-heat in the lower burner; Antiinflammation	36293	11.3	7.9
Shu-jing-huo-xue-tang	*Angelica sinensis*, *Paeonia lactiflora* Pall, *Glycyrrhiza uralensis* Fisch, *Rehmannia glutinosa* (Gaert.) Libosch, *Atractylodes lancea*, *Achyranthes bidentata* Blume, *Citrus tangerina pericarpium, Citrus reticulata* Blanco, *Prunus persica* (L.) Batsch, *Clematis chinensis* Osbeck, *Ligusticum striatum* DC., *Stephania tetrandra* S. Moore, *Notopterygium incisum*, *Angelica dahurica* (Fisch. ex Hoffm.) Benth, *Gentiana scabra* Bge, *Poria cocos*, *Zingiber officinale* Rosc.,	E: Relax the channels and activate blood; Also used in osteoarthritis and rheumatoid arthritis	32646	10.3	6.9
Xiang-sha-liu-jun-zi-tang	*Aquilaria sinensis*, *Amomum villosum* Lour, *Citrus reticulata* Blanco, *Pinellia ternata* (Thunb.), *Panax ginseng* C.A. Mey, *Poria cocos*, *Atractylodes macrocephala* Koidz., *Glycyrrhiza uralensis* Fisch.	E: Tonify and replenish qi Also used in functional dyspepsia and post-surgery colon cancer patients	28381	7.1	8.3
Gan-lu-yin	*Rehmannia glutinosa*, *Asparagus cochinchinensis* (Lour.) Merr., *Liriope spicata* (Thunb.) Lour., *Dendrobium nobile* Lindl., *Artemisia capillaris* Thunb, *Scutellaria baicalensis* Georgi, *Citrus aurantium* L., *Eriobotrya japonica* (Thunb.) Lindl., *Glycyrrhiza uralensis* Fisch,	H& E: Clear heat and nourish yin; Also used in Sicca syndrome	27563	6.1	7.6
Xue-fu-zhu-yu-tang	*Angelica sinensis* (Oliv.) Diels, *Ligusticum chuanxiong* hort, *Paeonia anomala* L., *Prunus persica* (L.), Batsch, *Carthamus tinctorius* L., *Rehmannia glutinosa* (Gaert.) Libosch, *Citrus aurantium* L., *Bupleurum chinense* DC., *Glycyrrhiza uralensis* Fisch, *Platycodon grandiflorum* (Jacq.) A. DC., *Achyranthes bidentata* Blume,	E: Promote blood circulation to remove blood stasis Also used in ischemic heart disease and hyperlipidemia.	27493	6.6	8.1
Du-huo-ji-sheng-tang	*Angelica pubescens* Maxim, *Taxillus chinensis* (DC.) Danser, *Eucommia ulmoides* Oliver, *Achyranthes bidentata* Blume, *Asarum heterotropoides* F. Schmidt, *Gentiana macrophylla* Pall, *Poria cocos*, *Cinnamomum cassia* Presl, *Saposhnikovia divaricata* (Turcz.) Schischk, *Ligusticum striatum* DC., *Panax ginseng* C.A. Mey., *Glycyrrhiza uralensis* Fisch, *Angelica sinensis*, *Paeonia lactiflora* Pall.	E: Reinforce the liver and kidney and tonify qi and blood; Also used in fracture, osteoarthritis and rheumatoid arthritis	27388	11.1	8.2
Suan- zao-ren-tang	*Ligusticum striatum* DC., *Ziziphus jujuba* Mill. *Poria cocos*, *Glycyrrhiza uralensis* Fisch, *Anemarrhena asphodeloides* Bge	E: Nourish blood to tranquilize the mind; Also used in insomnia and depression	26417	8	7.5
Ping-wei-san	*Citrus reticulata* Blanco, *Atractylodes lancea* (Thunb.), *Magnolia officinalis* Rehd, *Glycyrrhiza uralensis* Fisch	E: Activate the flow of Qi and regulate the stomach Also used in leukemia patients	26083	7.2	7.6

Note: *H* hepatic action, *E* extrahepatic action

## Discussion

This research is the first large-scale study on the utilization patterns of TCM by patients with HC and was conducted by analysing claims data from TCM and non-TCM clinic visits covered by the NHI in Taiwan. In a previous study [21], Chen et al. investigated the frequency and prescription patterns of Chinese herbal medicine for chronic hepatitis, including viral hepatitis and alcoholic hepatitis, and revealed the same three most common herbal formulas as in our study and a similar age group of patients, approximately in their 40s to 50s, seeking TCM. However, this study focused only on hepatitis C and demonstrated a different gender predominance and more details on comorbidities. According to Sievert’s review [22], the prevalence rate of hepatitis C is as high as 4 %, but the diagnosis rate is only 1.3 %, which is similar to our report. This discrepancy may be due to the fact that only symptomatic patients with HC would visit the hospital and have diagnostic records. Other HCV carriers without medical seeking behaviour would not be recorded in the national health insurance database.

Of the patients with HC, 66.4 % had previously used TCM. The acceptance of TCM among patients with HC is much greater in Taiwan than in other countries [11, 12]. In addition, approximately 16 % of the patients visited TCM clinics more than six times per year (Table 2). This high visiting frequency might be explained by the fact that many of these patients had chronic illnesses that required long-term care and treatment. Moreover, unlike the predisposition towards acupuncture in Europe [23], herbal remedies have been widely used in Taiwanese patients with HC (52.4 %). People in Taiwan believe that TCM can adjust the constitution of the human body, allowing small doses of herbal remedies to remain safe and suitable for long-term use [24]. Furthermore, the insurance coverage for TCM treatments might play a significant role in the high TCM usage in Taiwan [25]. As for receiving the current standard treatment, the rate of treatment in both arms was low (14 % and 17 %) in our study. This result is consistent with a previous nationwide survey in Taiwan (13.7 %) [26]. Although the anticipated treatment success rate is as high as 80 % in Taiwan, only 8.1 % of the population with HC achieved successful treatment. The major treatment barriers included fear of adverse effects, major disorders, ineligibility for insurance reimbursement, and lack of awareness of therapy.

Comparing the hazard ratio of comorbidities between TCM users and non-TCM users, the TCM users tended to have a lower risk (0.4 − 0.6 times) of hepatic diseases, suggesting a negative association of hepatitis B, liver cirrhosis, and alcoholic liver disease with TCM usage. One possible explanation for this finding is that the use of TCM might have a protective effect on liver diseases. Another explanation is that patients with impaired liver function would avoid herbal medications to prevent disease progression. The causal relationship warrants further research in the future. However, thyroid diseases, rheumatoid arthritis, osteoarthritis, and sicca syndrome were extrahepatic syndromes that had higher incidence rates in TCM users in Taiwan (Table 4). After adjusting for time of visit, Charlson comorbidity index score, and type of TCM service used, the hazard ratio of these comorbidities became less than 1 and was non-significant. This means the TCM-seeking behaviour is associated with extrahepatic diseases rather than hepatitis C. This finding might be attributed to the side effects of interferon-based antiviral therapy and the lack of satisfaction with the current conventional therapies [27].

To date, the NHIRD has collected diagnosis data via ICD-9-CM codes, which do not classify TCM syndromes or diagnosis. However, TCM prescriptions including formula or herbs are recorded in the NHIRD. By analysing the prescription patterns, we could obtain the possible TCM syndromes and indications for subjects with HC (Table 5). In our database, the most commonly used formulas and single herbs for HC were categorized into hepatic or extrahepatic based on their therapeutic action and clinical indications. Jia-Wei-Xiao-Yao-San, the most commonly used formula, has demonstrated pleiotropic effects in patients with HC, including anti-hepatic fibrosis [28], anti-hepatic cancer [29], anti-depressant [30, 31], and anti-hyperthyroidism effects [32]. However, it is unclear whether it has antiviral effects on HCV, and future investigations on this subject are warranted. Dan-Shen (*Salvia miltiorrhiza*), the most commonly used single herb, also has multiple hepatoprotective and extrahepatic effects, such as anti-hepatic fibrosis [33], anti-hepatic cancer [34, 35], anti-diabetic [36], and lipid-lowering [37] effects, but no apparent antiviral effects. Other commonly used prescriptions had similar multi-target effects, which implied that TCM physicians used these prescriptions to prevent disease progression or to relieve relevant extra-hepatic syndromes rather than to eradicate HCV.

The present study had some limitations. First, this study did not include therapies that were not covered by the NHI, such as newly antiviral agents or folk medicines [38], which were purchased directly from TCM herbal pharmacies. Consequently, the TCM utilization rates might have been underestimated. However, because only licensed TCM physicians can be reimbursed by the NHI system, the quality of the diagnoses and treatments in the NHIRD were ensured. Second, safety data in this retrospective study are lacking, and thus we cannot evaluate the safety of TCM. Third, our study only examined ambulatory visits to TCM or non-TCM clinics. Our results for visits to Western medical clinics, including inpatient services and emergency department visits, basically concurred with a previous study of outpatient visits [39]. TCM inpatient services, which mostly included hospital-based healthcare for senile populations, were not that popular and therefore only represented a small proportion of the TCM services received by HC patients.

## Conclusion

In summary, we conducted a nationwide, population-based study on the use of TCM in patients with HC based on one randomly selected cohort in 2010 from the NHIRD healthcare claims data in Taiwan. It is that more than 60 % of the TCM users were female and that the utilization of TCM increased with age and peaked in the age group of those 40 − 64 years old. Patients who had more extrahepatic diseases and were taking more antiviral agents tended to visit TCM clinics. Jia-Wei-Xiao-Yao-San and Dan-Shen (*Salvia miltiorrhiza*) were the most commonly used formula and single herb, with 88,124 person-days and 59,252 person-days, respectively. The high prevalence and distinct usage patterns of TCM in the Taiwanese HC population warrant more substantial, high-quality and/or well-designed clinical trials of TCM use.

## Abbreviations

CAM: Complementary and alternative medicine
HC: Hepatitis C
HCV: Hepatitis C virus
HR: Hazard ratio
ICD-9-CM: International Classification of Disease, 9th Revision, Clinical Modification
NCCIH: National Centre for Complementary and Integrative Health
NHI: National Health Insurance
NHIRD: National Health Insurance Research Database
TCM: Traditional Chinese Medicine
WHO: World Health Organization
